# Single-Session, Internet-Based Cognitive Behavioral Therapy to Improve Parenting Skills to Help Children Cope With Anxiety During the COVID-19 Pandemic: Feasibility Study

**DOI:** 10.2196/26438

**Published:** 2022-04-13

**Authors:** Tarja Korpilahti-Leino, Terhi Luntamo, Terja Ristkari, Susanna Hinkka-Yli-Salomäki, Laura Pulkki-Råback, Otto Waris, Hanna-Maria Matinolli, Atte Sinokki, Yuko Mori, Mami Fukaya, Yuko Yamada, Andre Sourander

**Affiliations:** 1 Research Centre for Child Psychiatry Department of Clinical Medicine, Faculty of Medicine University of Turku Turku Finland; 2 INVEST Child Psychiatry, INVEST Research Flagship Center Department of Clinical Medicine, Faculty of Medicine University of Turku Turku Finland; 3 Department of Child Psychiatry Turku University Hospital Turku Finland; 4 Department of Psychology and Human Developmental Sciences Graduate School of Education and Human Development Nagoya University Nagoya Japan; 5 Section of Medical Care and Consultation Nagoya City Central Care Center for Disabled Children Nagoya Japan

**Keywords:** adolescent, anxiety, child, cognitive behavioral therapy, coping, COVID-19, Internet, mental health, parents, web-based

## Abstract

**Background:**

The COVID-19 pandemic has had a major impact on families’ daily routines and psychosocial well-being, and technology has played a key role in providing socially distanced health care services.

**Objective:**

The first objective of this paper was to describe the content and delivery of a single-session, internet-based cognitive behavioral therapy (iCBT) intervention, which has been developed to help parents cope with children’s anxiety and manage daily situations with their children. The second objective was to report user adherence and satisfaction among the first participants who completed the intervention.

**Methods:**

The Let’s Cope Together intervention has been developed by our research group. It combines evidence-based CBT elements, such as psychoeducation and skills to manage anxiety, with parent training programs that strengthen how parents interact with their child and handle daily situations. A pre-post design was used to examine user satisfaction and the skills the parents learned. Participants were recruited using advertisements, media activity, day care centers, and schools and asked about background characteristics, emotional symptoms, and parenting practices before they underwent the iCBT. After they completed the 7 themes, they were asked what new parenting skills they had learned from the iCBT and how satisfied they were with the program.

**Results:**

Of the 602 participants who filled in the baseline survey, 196 (32.6%) completed the program’s 7 themes, and 189 (31.4%) completed the postintervention survey. Most (138/189, 73.0%) of the participants who completed the postintervention survey were satisfied with the program and had learned skills that eased both their anxiety (141/189, 74.6%) and their children’s anxiety (157/189, 83.1%). The majority (157/189, 83.1%) reported that they learned how to organize their daily routines better, and just over one-half (100/189, 53.0%) reported that the program improved how they planned each day with their children.

**Conclusions:**

The single-session iCBT helped parents to face the psychological demands of the COVID-19 pandemic. Future studies should determine how the participation rate and adherence can be optimized in digital, universal interventions. This will help to determine what kinds of programs should be developed, including their content and delivery.

## Introduction

There are considerable concerns about the possible short-term and long-term impact that the COVID-19 pandemic will have on the mental health of children and adolescents [1]. Families have faced substantial concerns and changes to their daily routines and social infrastructures during this global health emergency. These include worrying information and experiences, lockdowns and social distancing, school closures, and employment and economic uncertainties. Evidence suggests that children and adolescents have been experiencing feelings of fear, worry, sadness, anger, and loneliness, in addition to guilt about possibly spreading the virus [2]. Studies have shown that the pandemic has had a substantial impact on the well-being of parents and their children [3], and children and adolescents have reported a high prevalence of anxiety and depression [4]. Deteriorating mental health has been reported by adolescents and young adults in both clinical and community samples [5]. As parents play a significant role in how the crisis affects their children’s mental well-being, there is an increased, and urgent, need to provide help for parents. They need this so that they can communicate effectively with their children, ease their children’s anxiety, and maintain consistent routines that support the mental health of the whole family [1,6,7].

There is strong evidence that parental factors, such as warmth and modeling, influence children’s well-being, by decreasing anxiety [8]. According to a meta-analysis by Yap et al [9], preventive parenting interventions are important tools in reducing child internal problems and may have long-term effects on the child’s well-being. The COVID-19 crisis has challenged health care professionals to develop, and deliver, low-threshold interventions that reduce the adverse effects of the pandemic on the mental health of children. The social distancing restrictions that have been used to reduce the spread of the pandemic have made digital mental health interventions more acceptable [10].

Previous studies have shown that digitally delivered parent training, based on cognitive behavioral theory (CBT), has been effective in treating childhood anxiety [11] and disruptive behavior [12-14]. Randomized controlled trials of traditional face-to–face interventions have shown that brief, or single, CBT sessions have been effective in treating childhood anxiety [15] and posttraumatic stress [16]. A single-session, individually tailored, internet-based parenting intervention also improved self-reported parenting factors that are known to influence the development of depression and anxiety in adolescents [17]. These CBT sessions could be a suitable way to deliver basic methods that help parents to cope with anxiety and develop the skills they need during the COVID-19 pandemic. The great advantage of internet sessions is that they are remotely accessible and can be used anytime and anywhere.

There are already numerous online sources that provide psychoeducation for families on how to ease stress and anxiety during the pandemic, including resources provided by the World Health Organization [18] and UNICEF [19]. However, we are not aware of any research on universally delivered internet-based CBT (iCBT) interventions that focus on improving parental skills to help children to cope with anxiety during the COVID-19 pandemic. That is why our research group has developed a single-session iCBT for parents. This new initiative is based on our considerable expertise in developing, evaluating, and implementing evidence-based, digital interventions that provide parental training for children with behavioral problems and reduce children’s anxiety [12-14]. The aim of this paper was to describe the content and delivery of the Let’s Cope Together intervention and report background characteristics, user adherence, and satisfaction levels of the first participants.

## Methods

### Description of the Intervention

**Figure 1:**
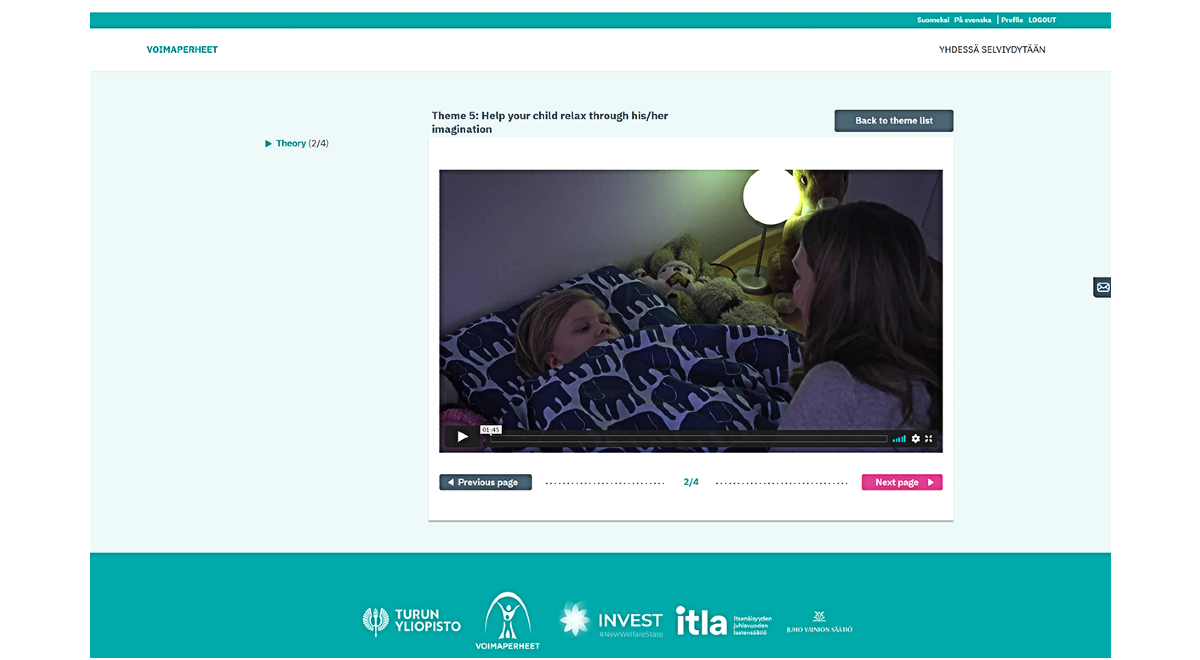
Example of the online material, in which positive mental imagery helps children to manage anxiety [20].

Let’s Cope Together is a universal, single-session iCBT that is intended for all parents with children and adolescents under the age of 18 years in Finland who have access to a digital device with an internet connection. The aim of the intervention is to use this simple, digitally delivered program to improve parental skills, so that they can help their children to cope with any anxiety during the COVID-19 pandemic. The intervention, which is based on CBT and the principles of positive parenting, consists of various online materials (Figure 1).

**Figure 2:**
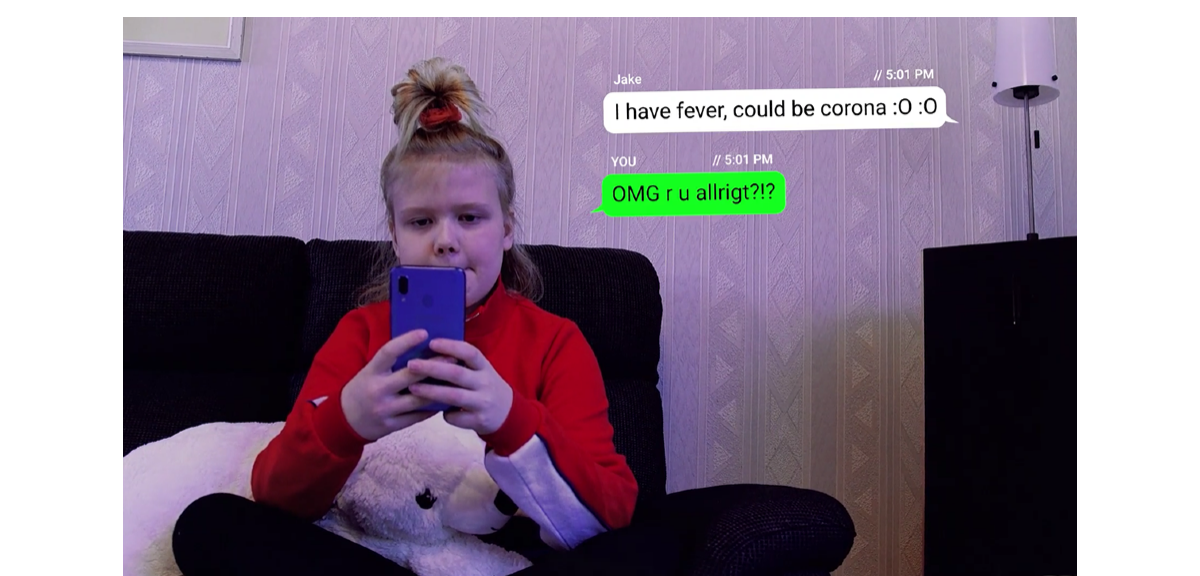
Still from a video that forms part of the program.

The intervention was developed in March 2020 and April 2020 by a multidisciplinary team at the University of Turku, Finland. The team included CBT therapists, child psychiatrists, information technology personnel, and experienced family coaches with special training in delivering digital interventions. The content of the program has been specifically tailored to deal with the current COVID-19 pandemic. It includes evidence-based CBT elements that can be used for any kind of anxiety, such as psychoeducation, cognitive restructuring, and relaxation techniques, as well as strategies specifically targeting COVID-19–related anxiety (Figure 2). Parents who go through the program learn how to manage anxiety, by using techniques such as deep breathing. They also learn mental practice techniques that strengthen positive thinking and encourage children to display positive behavior. Additional elements from our existing CBT-based parent training programs have also been included, which strengthen parent-child interactions, manage daily transitions, and help parents plan their day with their child. Table 1 provides an overview of the iCBT content.

**Figure 3:**
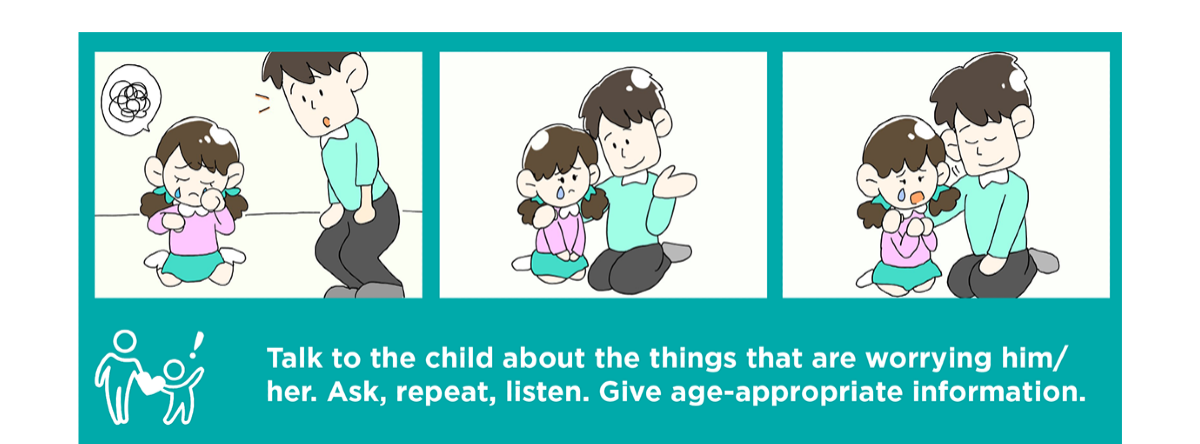
One of the program’s manga-style comic illustrations.

The program contains 7 themes that the parents can work through at their own pace. They can complete the intervention in one go or by logging in and out several times. Distinct themes are used to clarify the content, and each theme is comprised of 4 to 5 pages. Our aim is to motivate parents by presenting the psychoeducation and skill training content of the program in a variety of ways. The total program is comprised of 29 pages of text, 6 videos, 2 audio clips, and 5 manga-style cartoons. The text components are short, simple, and easy to read and are supported by the manga cartoons, which illustrate the key points of each theme (Figure 3). The videos have been included to show how the skills work in practice. There are also links to additional information on each topic.

The intervention is delivered via the internet using a secure TLS-encrypted connection. The website is located on a server maintained by the University of Turku and complies with the university’s information security requirements and data protection regulations.

The content of the program was planned by our multidisciplinary team, which previously developed and studied an iCBT for childhood anxiety and a parent training program for behavioral problems. In the midst of the rapidly worsening pandemic in spring 2020, we needed to decide how much information, and what kind of content, a single-session intervention should contain. In addition, we had to take account of the stressful situations that the families who would use the program were facing. The team chose to focus on providing psychoeducation and basic coping skills needed to manage anxiety based on CBT theory and practice. This program also builds on our previous, targeted parent training intervention [13,14]. Our existing experience in this area enabled us to choose the parenting skills that would be most effective in helping *families* to cope with everyday life during the ongoing pandemic. Because the Let’s Cope Together program is an unguided digital intervention, we only chose those components that do not require professional guidance. For example, we excluded the reward system featured in our previous internet-based intervention. The core challenge was to make the content as concise as possible. In addition, the content was primarily developed for younger school-aged children, and it was not possible to adjust the content so that it was optimized for all age groups, such as adolescents.

**Table 1:** Content of the Let’s Cope Together single-session, internet-based cognitive behavioral therapy (iCBT) intervention

Theme	Content
Theme 1. Introduction	How to use the program
Theme 2. Identifying anxiety	Psychoeducation about anxiety: What are normal worries and fears, and how does anxiety manifest in children´s behavior? Bodily sensations and thinking, anxiety in different age groups and when to seek additional help
Theme 3. Listening to your child	How to talk to children about COVID-19 and their fears about it; how parents can calm themselves down and provide a suitable environment before talking with their child; how to ask open questions, listen, and validate their child’s feelings
Theme 4. Relaxing by deep breathing	How parents can learn a deep-breathing technique and teach it to their child, so that they can both learn how to relax
Theme 5. Using mental practice techniques	Instructions on how to use positive mental practice techniques; how parents can teach their child to create a safe, imaginary place by using all of their senses.
Theme 6. Strengthening positive thinking	What are negative thoughts, and how do they affect feelings and behavior? How to recognize them and turn negative thoughts into positive ones
Theme 7. Positive parenting skills	Positive parenting strategies; how to plan daily routines and manage daily transitional situations; how to plan activities with children, motivate them, and give them positive feedback

### Study Design

There was an urgent need to initiate the study as quickly as possible due to the pandemic. The data were collected between April 17, 2020 and June 1, 2020. The pandemic lockdown started in Finland on March 16, 2020. Schools were closed, hobbies were discontinued, parents worked at home if they could, and schoolwork was carried out remotely. Families were asked not to use day care. A state of emergency was declared, which legalized additional local restrictions.

The participants were recruited through a multimedia advertisement and press release campaign, which included national media, social media—mainly Facebook and Instagram—and digital news. We also asked schools, school nurses, day care providers, and health care personnel to spread the word. There were no specific inclusion and exclusion criteria. Although the program was primarily developed for parents, other adults taking care of children, such as grandparents, other family members, and foster parents, could take part. We asked for the participants’ relationship to the child before they entered the program and the study. Professionals working with children were also encouraged to use the content of the program to support their own work. They were able to get a code from the study group so that they could enter the program without completing any questions.

A pre-post design was used. After the participants completed the digital, informed consent form, they were directed to the baseline assessment, which asked them for background information and details of the child’s and parents’ emotional symptoms and parenting skills. Once they completed this, they could proceed to the intervention. After they had completed the intervention, we asked them about their general satisfaction with the program and whether they felt the program would improve their parenting skills. The participants could contact a member of the research group by phone or email if they wanted further information at any stage of the study.

### Measures

The background information we requested before the intervention included the participant’s age and level of education, the number of adults who played a parenting role in the child’s life, the child’s gender, and whether they attended school or day care. We also asked about any emotional symptoms they and their child were experiencing, including sleep problems, somatic symptoms, restlessness and worries, fears, or sadness (Multimedia Appendix 1). The responses ranged from 0 for “not at all” to 5 for “all the time,” and the maximum scores were 25 for the child and 20 for the parents. The questions were specifically formulated for this study. They asked the participants about symptoms that are generally included in validated surveys on anxiety and depression, such as the Strengths and Difficulties Questionnaire and the Screen for Child Anxiety Related Disorders [21,22]. It was not possible to use full surveys or their subscales, as we decided to use as few questions as possible. No specific data were collected regarding the reason for the anxiety. The parenting skills included in the pre-intervention survey were related to the content of the intervention and included 12 items on how well the parent could notice the child’s anxiety, act as a positive role model, cope with their own anxiety and the child’s anxiety, manage daily routines, and plan ahead with the child. The answers ranged from 0 for “strongly disagree” to 6 for “strongly agree,” and the maximum score was 72. Cronbach alpha was used to measure the internal consistency of the parenting skills and the child’s and the parent’s emotional symptoms, and these values were 0.86, 0.81, and 0.79, respectively.

After they finished the program, we asked the parents 3 general questions about how satisfied they were with the program and whether they would recommend it to others. They were also asked 6 questions about whether they had learned new skills about how to identify their child’s anxiety, act as a positive role model, cope with their own anxiety and their child’s anxiety, manage daily routines, and plan ahead with the child.

### Statistical Analyses

The baseline survey was completed by 602 participants (524/602 [87.0%] of them were parents), and 189 (31.4%) also completed the postprogram survey.

We studied the potential differences in baseline measures between those who completed the intervention and those who did not. Pearson chi-square tests or Fisher exact tests were used for categorical variables, and Student *t* tests were used for continuous variables. Statistical significance was based on a 2-sided *P*<.05. All the statistical analyses were performed with SAS version 9.4 (SAS Institute, Cary, NC).

### Ethical Issues

The Ethics Committee of the Hospital District of Southwest Finland approved the study (Turku University Hospital Ethical Board Journal number: 14/1801/2020). Participation in the study was voluntary and could be discontinued at any time. The requirements relating to information security were considered carefully when collecting, storing, analyzing, and archiving the data. Research codes were used, and no individuals could be identified from the results.

## Results

About 9000 individuals visited the study website, 602 filled in the baseline survey, 468 (468/602, 77.7%) started the intervention, and 196 (196/602, 32.6%) completed all 7 themes. The postintervention survey was completed by 189 (189/602, 31.4%) of the participants who completed the baseline survey (Table 2).

We found that 77 (77/189, 40.7%) participants went through the material by logging in just once, 46 (46/189, 24.3%) by logging in twice, 34 (34/189, 18.0%) by logging in 3 times, and 32 (32/189, 16.9%) by logging in 4 times or more. The mean time that it took to complete the whole program was 73 minutes.

**Table 2:** Participants who completed each phase of the program and the study.

Sample	Baseline, n (%)	iCBT^a^ themes (1-7)^b^, n (%)								After iCBT, n (%)
		1	2	3	4	5	6	7		
Number of completers	602 (100)	412 (68.4)	378 (62.8)	340 (56.5)	283 (47.0)	240 (39.9)	214 (35.5)	196 (32.6)	189 (31.4)	

^a^iCBT: internet-based cognitive behavioral therapy.

^b^The participants could only proceed to the next theme or the postintervention survey if all the previous parts of the study were completed.

There were no significant differences between those who finished the study and those who discontinued at some point after filling in the baseline survey, when it came to education level, family structure, emotional symptoms, or self-reported parenting skills (Multimedia Appendix 2). The same was true for the child’s gender, child’s emotional symptoms, and whether the child attended day care or school. The mean age of the participants was slightly higher for those who finished the study than those who did not (42.2 years vs 40.4 years), and the participants were more likely to be another caregiver, rather than a parent (30/189, 15.9% vs 32/413, 7.8%). In addition, sensitivity analyses showed no significant differences in any of the background factors between those who finished the study and those who discontinued at some point after filling in the baseline survey. This included the analysis that just comprised parents.

Almost three-quarters (138/189, 73.0%) of those who completed the intervention and postintervention survey were satisfied with the program, and even more (153/189, 81.0%) reported that they would recommend the program to others (Table 3).

Most of those who finished the postintervention survey reported that they learned about childhood anxiety (146/189, 77.2%) and the skills they needed to calm themselves (141/189, 74.6%) and their child (157/189, 83.1%). In addition, 48.7% (92/189) of them reported that they had learned how to organize their daily routines better, and 53.0% (100/189) reported that they had learned how to improve the way they planned days with their child. The results remained almost the same when the sensitivity analyses focused on just parents rather than all participants.

**Table 3:** Participants’ satisfaction with the intervention and whether they learned new skills (n=189).

Questionnaire items			Agree, n (%)		Do not agree or disagree, n (%)		Disagree, n (%)		Missing, n (%)
**General satisfaction**									
	Program was easy to use	166 (87.8)		14 (7.4)		2 (1.1)		7 (3.7)	
	Was satisfied with the program	138 (73.0)		37 (19.6)		7 (3.7)		7 (3.7)	
	Would recommend the program to others	153 (81.0)		24 (12.7)		5 (2.7)		7 (3.7)	
**Developed better skills with regard to:**									
	Knowledge about their child’s anxiety	146 (77.2)		23 (12.2)		13 (6.9)		7 (3.7)	
	How to show a positive example	152 (80.5)		27 (14.3)		3 (1.6)		7 (3.7)	
	Ways to calm themselves down	141 (74.6)		27 (14.3)		14 (7.4)		7 (3.7)	
	Ways to calm the child down	157 (83.1)		16 (8.5)		9 (4.7)		7 (3.7)	
	Organizing daily routines	92 (48.7)		65 (34.4)		25 (13.2)		7 (3.7)	
	Planning days together with the child	100 (53.0)		64 (33.9)		18 (9.5)		7 (3.7)	

## Discussion

### Principal Findings

To our knowledge, this is the first paper to describe a universal digital parenting intervention that aims to improve parental skills so that they can help their children to cope with anxiety during the COVID-19 pandemic. In general, single-session interventions have been shown to be effective in treating anxiety, conduct problems, and posttraumatic stress disorders in children and adolescents [15,16,23]. Single-session, digitalized parenting interventions have also been effective in improving self-reported parenting practices [17]. Most of the parents who completed both the Let’s Cope Together program and postintervention survey were satisfied with the intervention and would recommend it to others. Most parents also reported that they had acquired knowledge about their children’s anxiety and had learned skills to calm themselves and their child down. Half of the parents reported that they learned how to organize daily routines and plan ahead better with their child.

The number of participants in this study was rather low, despite our substantial efforts to inform the target population about the study through online resources, including social media and digital news, schools, and day care centers. About 9000 individuals visited the study website, but only 602 registered and completed the baseline questions. Of these, about one-third finished the program and answered the postintervention questions.

There are a number of possible explanations for the low number of participants. Numerous other channels have provided support to help children and parents handle the psychosocial stress of the pandemic. These include digital news, interviews, and blogs that provide advice from experts, such as psychologists or psychotherapists. There are also several chat and telephone services run by third-sector organizations. It is likely that asking participants to provide personal data may have significantly decreased the number of participants, especially as there have been other sources of anonymous help. In addition, some of the services that were available also included human guidance, which was not the case with our program. The pandemic started relatively late in Finland, in comparison to a number of European countries, which meant that the government had more time to prepare. As a result, the number of people who have had the virus, been hospitalized, or died has been among the lowest in the world, as a proportion of the whole population. It is possible that the controlled situation in Finland reduced the need for help and that was reflected in the low participation rates.

It has been generally acknowledged that there are low participation rates in universal interventions. For example, Sampaio et al [24] found that less than 15% of the people who were eligible to take part in a universal program for the parents of preschoolers participated. Another study, by March et al [25], found that about 20% of those that logged on to a website that provided iCBT for youth anxiety did not start the program and about 30% dropped out during the first 2 sessions. It is also common for participants to fail to complete all modules of universal programs, for example due to a lack of time, technical problems, or no need for the support provided by the intervention [26].

There are some important questions that need to be answered, including who participates in programs and who benefits the most from them. It is understandable that families who do not experience the problems that the intervention tackles are not motivated to participate. In the present study, we did not find differences between those who did and did not finish the program, with regards to emotional symptoms, self-reported parenting skills, or sociodemographic characteristics. However, we were only able to study those who filled in the baseline questionnaire*.* The average baseline scores for emotional symptoms and self-reported parenting skills could be considered quite good already, and it might be that high-functioning families were over-represented. It is likely that parents who are already aware of, and able to think about, their children’s feelings may be more motivated to take part in digital, universal programs that require initiative and active participation. At the same time, lower functioning families could need more direct human motivation and support, which was not available in the present study, due to an uncontrollable number of potential participants. Further research is needed on the importance of human guidance with regard to how effective digital interventions are among different groups of participants. In addition, the characteristics of the participating parents might have had an impact on the completion rates in the present study. For example, the iCBT was based on material that was primarily developed by our study group for children under 13 years of age. It may be possible that this younger focus made the parents of adolescents less motivated to complete the program and the study.

At the same time, universal, digital interventions with no human guidance may be cost-effective when they are delivered to those who are willing to participate and able to benefit from the intervention. That is why it is crucial to study how the content and delivery of interventions should be developed, so that they respond to the individual needs of different families. However, the pressure posed by the worsening pandemic, during the spring 2020 development phase, meant that we were not able to study the needs and preferences of the end users, with regard to both the content and delivery methods, before the iCBT was launched. It might have had positive effect on the participation rate if we had been able to do this.

### Strengths and Limitations

The limitations of the study included the pre-post test design instead of a randomized controlled trial and the limited number of questions that were specifically developed for the present study. As this was a feasibility study, we only measured general satisfaction and the participants’ experience with learning new skills straight after the program. As the pandemic eases, we will report further feedback, including follow-up results on changes in anxiety symptoms. Additionally, we did not have specific inclusion and exclusion criteria. The program was developed for parents, but other adults caring for children could take part. It is possible that participants, particularly those who completed the posttest, were more interested and motivated to learn new skills and more satisfied with the program. These issues limit the interpretation and generalizability of the findings. However, the study design was simple and nonselective and contained a limited number of short questions to encourage participation. The strengths of the study included the considerable previous experience of the multidisciplinary team that developed the intervention. This included CBT therapists, child psychiatrists, information technology personnel, and experienced family coaches with special training in delivering digital interventions.

### Conclusion

The internet-based Let’s Cope Together single-session parenting intervention helped families to cope with parenting demands during the COVID-19 pandemic. It is likely that we could face similar pandemics or other global crises in the near future. Developing and researching the effectiveness and implementation of universal digital interventions provide important preparation for managing future crises. Enhancing parenting skills is an important goal, as it increases the resilience of families with young children. The impact of current and future crises on mental health is substantial, particularly for those who have been affected most by the current pandemic. These include those with mental health and parenting problems, children and adolescents, immigrants, and frontline staff. Digitally delivered, evidence-based interventions can provide inexpensive, low-threshold help for large populations when face-to-face interventions are unavailable or not feasible.

## Appendix

Multimedia Appendix 1 Questionnaire administered to the parents at baseline.

Multimedia Appendix 2 Frequencies and differences in background factors of those who did and did not complete the baseline and postintervention survey.

## Abbreviations

iCBT: internet-based cognitive behavioral therapy

## References

[1] Golberstein E, Wen H, Miller BF (2020). Coronavirus disease 2019 (COVID-19) and mental health for children and adolescents. JAMA Pediatr.

[2] Idoiaga Nahia, Berasategi Naiara, Eiguren Amaia, Picaza Maitane (2020). Exploring children's social and emotional representations of the COVID-19 pandemic. Front Psychol.

[3] Patrick SW, Henkhaus LE, Zickafoose JS, Lovell K, Halvorson A, Loch S, Letterie M, Davis MM (2020). Well-being of parents and children during the COVID-19 pandemic: a national survey. Pediatrics.

[4] Zhou S, Zhang L, Wang L, Guo Z, Wang J, Chen J, Liu M, Chen X, Chen J (2020). Prevalence and socio-demographic correlates of psychological health problems in Chinese adolescents during the outbreak of COVID-19. Eur Child Adolesc Psychiatry.

[5] Hawke LD, Barbic SP, Voineskos A, Szatmari P, Cleverley K, Hayes E, Relihan J, Daley M, Courtney D, Cheung A, Darnay K, Henderson JL (2020). Impacts of COVID-19 on youth mental health, substance use, and well-being: a rapid survey of clinical and community samples. Can J Psychiatry.

[6] Dalton L, Rapa E, Stein A (2020). Protecting the psychological health of children through effective communication about COVID-19. Lancet Child Adolesc Health.

[7] Fegert JM, Vitiello B, Plener PL, Clemens V (2020). Challenges and burden of the Coronavirus 2019 (COVID-19) pandemic for child and adolescent mental health: a narrative review to highlight clinical and research needs in the acute phase and the long return to normality. Child Adolesc Psychiatry Ment Health.

[8] Yap MBH, Jorm AF (2015). Parental factors associated with childhood anxiety, depression, and internalizing problems: a systematic review and meta-analysis. J Affect Disord.

[9] Yap MBH, Morgan AJ, Cairns K, Jorm AF, Hetrick SE, Merry S (2016). Parents in prevention: A meta-analysis of randomized controlled trials of parenting interventions to prevent internalizing problems in children from birth to age 18. Clin Psychol Rev.

[10] Wind TR, Rijkeboer M, Andersson G, Riper H (2020). The COVID-19 pandemic: The 'black swan' for mental health care and a turning point for e-health. Internet Interv.

[11] Vigerland S, Ljótsson B, Thulin U, Öst LG, Andersson G, Serlachius E (2016). Internet-delivered cognitive behavioural therapy for children with anxiety disorders: A randomised controlled trial. Behav Res Ther.

[12] Sourander A, McGrath PJ, Ristkari T, Cunningham C, Huttunen J, Lingley-Pottie P, Hinkka-Yli-Salomäki S, Kinnunen M, Vuorio J, Sinokki A, Fossum S, Unruh A (2016). Internet-assisted parent training intervention for disruptive behavior in 4-year-old children: a randomized clinical trial. JAMA Psychiatry.

[13] Sourander A, McGrath PJ, Ristkari T, Cunningham C, Huttunen J, Hinkka-Yli-Salomäki S, Kurki M, Lingley-Pottie P (2018). Two-year follow-up of internet and telephone assisted parent training for disruptive behavior at age 4. J Am Acad Child Adolesc Psychiatry.

[14] Ristkari T, Kurki M, Suominen A, Gilbert S, Sinokki A, Kinnunen M, Huttunen J, McGrath P, Sourander A (2019). Web-based parent training intervention with telephone coaching for disruptive behavior in 4-year-old children in real-world practice: implementation study. J Med Internet Res.

[15] Cobham VE, Filus A, Sanders MR (2017). Working with parents to treat anxiety-disordered children: A proof of concept RCT evaluating Fear-less Triple P. Behav Res Ther.

[16] Haag A, Landolt MA, Kenardy JA, Schiestl CM, Kimble RM, De Young AC (2020). Preventive intervention for trauma reactions in young injured children: results of a multi-site randomised controlled trial. J Child Psychol Psychiatry.

[17] Cardamone-Breen MC, Jorm AF, Lawrence KA, Rapee RM, Mackinnon AJ, Yap MBH (2018). A single-session, web-based parenting intervention to prevent adolescent depression and anxiety disorders: randomized controlled trial. J Med Internet Res.

[18] Coronavirus disease (COVID-19) advice for the public: Advocacy. World Health Organization.

[19] Coronavirus (COVID-19) parenting tips. UNICEF.

[20] (2020). Safeplace. Vimeo.

[21] Birmaher B, Khetarpal S, Brent D, Cully M, Balach L, Kaufman J, Neer SM (1997). The Screen for Child Anxiety Related Emotional Disorders (SCARED): scale construction and psychometric characteristics. J Am Acad Child Adolesc Psychiatry.

[22] Goodman R (1999). The extended version of the Strengths and Difficulties Questionnaire as a guide to child psychiatric caseness and consequent burden. J Child Psychol Psychiatry.

[23] Schleider JL, Weisz JR (2017). Little treatments, promising effects? Meta-analysis of single-session interventions for youth psychiatric problems. J Am Acad Child Adolesc Psychiatry.

[24] Sampaio F, Sarkadi A, Salari R, Zethraeus N, Feldman I (2015). Cost and effects of a universal parenting programme delivered to parents of preschoolers. Eur J Public Health.

[25] March S, Spence SH, Donovan CL, Kenardy JA (2018). Large-scale dissemination of internet-based cognitive behavioral therapy for youth anxiety: feasibility and acceptability study. J Med Internet Res.

[26] Morgan AJ, Rapee RM, Salim A, Goharpey N, Tamir E, McLellan LF, Bayer JK (2017). Internet-delivered parenting program for prevention and early intervention of anxiety problems in young children: randomized controlled trial. J Am Acad Child Adolesc Psychiatry.

