# Supplementary material for: Single-Session, Internet-Based Cognitive Behavioral Therapy to Improve Parenting Skills to Help Children Cope With Anxiety During the COVID-19 Pandemic: Feasibility Study
Source: J Med Internet Res. 2022 Apr 13;24(4):e26438. doi: 10.2196/26438 (PMC9009379; doi:10.2196/26438)
Supplement: Multimedia Appendix 1 [file jmir_v24i4e26438_app1.pdf]

Multimedia Appendix 1. Questionnaire administered to the parents at baseline.

We would like to ask you what you have done with your child during the last week. Choose the option that suits you best for each statement.

1. I have created daily routines that suit me and my child.
   - ☐ Fully agree
   - ☐ Agree
   - ☐ Agree somewhat
   - ☐ Neither agree nor disagree
   - ☐ Disagree somewhat
   - ☐ Disagree
   - ☐ Fully disagree

2. I plan the day ahead of time with my child.
   - ☐ Fully agree
   - ☐ Agree
   - ☐ Agree somewhat
   - ☐ Neither agree nor disagree
   - ☐ Disagree somewhat
   - ☐ Disagree
   - ☐ Fully disagree

3. I know how to act as a positive example for my child.
   - ☐ Fully agree
   - ☐ Agree
   - ☐ Agree somewhat
   - ☐ Neither agree nor disagree
   - ☐ Disagree somewhat
   - ☐ Disagree
   - ☐ Fully disagree

4. I have ways to calm myself down.
   - ☐ Fully agree
   - ☐ Agree
   - ☐ Agree somewhat
   - ☐ Neither agree nor disagree
   - ☐ Disagree somewhat
   - ☐ Disagree
   - ☐ Fully disagree

5. I have ways to calm my child down.
   - ☐ Fully agree
   - ☐ Agree
   - ☐ Agree somewhat
   - ☐ Neither agree nor disagree
   - ☐ Disagree somewhat
   - ☐ Disagree
   - ☐ Fully disagree

6. I can identify when my child is anxious.
   - ☐ Fully agree
   - ☐ Agree
   - ☐ Agree somewhat
   - ☐ Neither agree nor disagree
   - ☐ Disagree somewhat
   - ☐ Disagree
   - ☐ Fully disagree

7. I know what to do when my child seems anxious.
   - ☐ Fully agree
   - ☐ Agree
   - ☐ Agree somewhat
   - ☐ Neither agree nor disagree
   - ☐ Disagree somewhat
   - ☐ Disagree
   - ☐ Fully disagree

8. When my child is worried, I focus on asking what is worrying him/her and listening to the answer.
   - ☐ Fully agree
   - ☐ Agree
   - ☐ Agree somewhat
   - ☐ Neither agree nor disagree
   - ☐ Disagree somewhat
   - ☐ Disagree
   - ☐ Fully disagree

9. When my child is anxious, I know to guide her/him to use her/his imagination in order to feel safer.
   - ☐ Fully agree
   - ☐ Agree
   - ☐ Agree somewhat
   - ☐ Neither agree nor disagree
   - ☐ Disagree somewhat
   - ☐ Disagree
   - ☐ Fully disagree

10. I use breathing exercises to calm my child.
   - ☐ Fully agree
   - ☐ Agree
   - ☐ Agree somewhat
   - ☐ Neither agree nor disagree
   - ☐ Disagree somewhat
   - ☐ Disagree
   - ☐ Fully disagree

11. When my child is anxious, I encourage him/her to think positive thoughts.
   - ☐ Fully agree
   - ☐ Agree
   - ☐ Agree somewhat

- ☐ Neither agree nor disagree
- ☐ Disagree somewhat
- ☐ Disagree
- ☐ Fully disagree

12. I believe that I can make my child feel safe.
    - ☐ Fully agree
    - ☐ Agree
    - ☐ Agree somewhat
    - ☐ Neither agree nor disagree
    - ☐ Disagree somewhat
    - ☐ Disagree
    - ☐ Fully disagree

Below we have described symptoms that many people are likely to suffer from at the moment. Choose the option that best describes how you have been feeling during the past week.

1. I have trouble sleeping (difficulties falling asleep, waking up during the night or nightmares)
    - ☐ All the time
    - ☐ Nearly all the time
    - ☐ Often
    - ☐ Sometimes
    - ☐ Rarely
    - ☐ Not at all

2. I suffer from physical symptoms such as heart palpitations, dizziness or headaches.
    - ☐ All the time
    - ☐ Nearly all the time
    - ☐ Often
    - ☐ Sometimes
    - ☐ Rarely
    - ☐ Not at all

3. I am feeling anxious.
    - ☐ All the time
    - ☐ Nearly all the time
    - ☐ Often
    - ☐ Sometimes
    - ☐ Rarely
    - ☐ Not at all

4. I feel down.
    - ☐ All the time
    - ☐ Nearly all the time
    - ☐ Often
    - ☐ Sometimes
    - ☐ Rarely
    - ☐ Not at all

The following questions relate to your child. Assess your situation in the past week.

1. My child seems worried.
   - ☐ All the time
   - ☐ Nearly all the time
   - ☐ Often
   - ☐ Sometimes
   - ☐ Rarely
   - ☐ Not at all

2. My child has trouble sleeping (difficulties falling asleep, waking up during the night or nightmares)
   - ☐ All the time
   - ☐ Nearly all the time
   - ☐ Often
   - ☐ Sometimes
   - ☐ Rarely
   - ☐ Not at all

3. My child is frightened.
   - ☐ All the time
   - ☐ Nearly all the time
   - ☐ Often
   - ☐ Sometimes
   - ☐ Rarely
   - ☐ Not at all

4. My child suffers from physical symptoms, such as stomach ache or headaches.
   - ☐ All the time
   - ☐ Nearly all the time
   - ☐ Often
   - ☐ Sometimes
   - ☐ Rarely
   - ☐ Not at all

5. My child is anxious.
   - ☐ All the time
   - ☐ Nearly all the time
   - ☐ Often
   - ☐ Sometimes
   - ☐ Rarely
   - ☐ Not at all
