# Supplementary material for: Single-Session, Internet-Based Cognitive Behavioral Therapy to Improve Parenting Skills to Help Children Cope With Anxiety During the COVID-19 Pandemic: Feasibility Study
Source: J Med Internet Res. 2022 Apr 13;24(4):e26438. doi: 10.2196/26438 (PMC9009379; doi:10.2196/26438)
Supplement: Multimedia Appendix 2 [file jmir_v24i4e26438_app2.docx]

**Multimedia Appendix 2.**

**Table S1.** Frequencies and differences in background factors of those who did and did not complete the baseline and post-intervention survey

| Participant characteristics | Not completed (n=413) | Completed (n=189) | *P* value |
| --- | --- | --- | --- |
| **Participant** / n (%) | | | .02 |
| Mother | 346 (83.8) | 143 (75.7) |  |
| Father | 27 (6.5) | 10 (5.3) |  |
| Other | 32 (7.8) | 30 (15.9) |  |
| Missing | 8 (1.9) | 6 (3.2) |  |
| **Age** / mean (SD) | 40.4 (7.0) | 42.2 (8.2) | .008 |
| **Participant’s education** / n (%) | | | .37 |
| Basic | 8 (1.9) | 2 (1.1) |  |
| Lower secondary | 110 (26.7) | 41 (21.7) |  |
| Upper secondary | 287 (69.5) | 140 (74.1) |  |
| Missing | 8 (1.9) | 6 (3.2) |  |
| **Family structure** /n (%) | | | .12 |
| Two adults | 307 (76.4) | 133 (72.7) |  |
| One adult | 67 (16.7) | 27 (14.8) |  |
| Other | 6 (1.5) | 3 (1.6) |  |
| No children^a^ | 22 (5.5) | 20 (10.9) |  |
| **Emotional** **symptoms** /mean (SD)^b^ | 8.6 (3.2) | 8.4 (2.9) | .38 |
| **Parenting skills** / mean (SD)^c^ | 51.3 (9.1) | 52.1 (7.4) | .31 |
| Child characteristics | | | |
| **Child’s gender** /n (%) | | | .89 |
| Boy | 336 (52.01) | 135 (52.1) |  |
| Girl | 308 (47.7) | 124 (47.9) |  |
| Other | 2 (0.3) | 0 (0.0) |  |
| **Where child was during weekdays /** n (%) | | | .11 |
| At school | 63 (7.0) | 20 (7.7) |  |
| Doing schoolwork at home | 516 (57.6) | 146 (55.9) |  |
| In day care | 98 (10.9) | 20 (7.7) |  |
| Under school age at home | 209 (23.3) | 67 (25.7) |  |
| Missing responses | 10 (1.1) | 8 (3.1) |  |
| **Child’s emotional symptoms** / mean (SD)^d^ | 9.8 (3.6) | 10.0 (3.8) | .57 |

*P* value refers to the possible difference between the background factors, emotional symptoms and self-reported parenting skills of the two groups. ^a^ Represents people working with children. ^b^ Scores out of 20, with higher scores indicating more symptoms. ^c^ Scores out of 72, with higher scores indicating better skills. ^d^ Scores out of 25, with higher scores indicating more symptoms.
